# DLC-1 is an independent prognostic marker and potential therapeutic target in hepatocellular cancer

**DOI:** 10.1186/s13000-016-0470-x

**Published:** 2016-02-04

**Authors:** L. J. Song, Q. Liu, X. R. Meng, SH. L Li, L. X. Wang, Q. X. Fan, X. Y. Xuan

**Affiliations:** Department of Oncology, the first affiliated hospital of Zhengzhou University, Henan, 450000 China; Department of Neurosurgery, the fifth affiliated hospital of Zhengzhou University, Henan, 450000 China; Department of Pathology, the first affiliated hospital of Zhengzhou University, Henan, 450000 China; Department of Microbiology and Immunology, Basic Medical School of Zhengzhou University, 100 Kexue Road, Zhengzhou, Henan 450001 China

**Keywords:** HCC, DLC-1, Rho A, ROCK2, moesin

## Abstract

**Background:**

The 5-year survival rate of patients with hepatocellular cancer (HCC) was very low because of invasion and metastasis in the early stage. Biomarkers might help predict early occurrence of invasion and metastasis. Accumulating evidence has shown that deleted in liver cancer-1 (DLC1) may be considered as a metastasis suppressor gene in numerous solid and hematological cancers. However, its prognostic role and mechanisms that regulate and coordinate these activities remain poorly understood.

**Methods:**

With the method of immunohistochemistry, the expression of DLC-1 as well as Rho A, ROCK2, moesin had been characterized in 80 HCC tissues and adjacent noncancerous tissues. The correlation between their expression and their relationships with clinicopathological characteristics of HCC were also investigated. In addition, the prognostic value of DLC1 expression within the tumor tissues was assessed by Cox regression and Kaplan-Meier analysis.

**Results:**

DLC1 expression was significantly lower in HCC tissues than in adjacent noncancerous tissues, and DLC-1 expression was found to be negatively correlated with tumor differentiation, TNM stage and lymph node metastasis. Furthermore, DLC-1 expression was found to inversely correlate with Rho A, ROCK2 and moesin which were all highly expressed in HCC tissues. Kaplan-Meier analysis showed that significantly longer 5-year survival rate was seen in HCC patients with higher DLC1 expression, compared to those with lower expression of DLC1. Multivariate Cox proportional hazard analyses revealed that DLC1 was an independent factor affecting the overall survival probability.

**Conclusion:**

DLC1 could be served as a tumor suppressor gene in the progression especially in the invasion and metastasis of HCC. DLC1 perhaps played its role by regulating the expression of Rho A, ROCK2 and moesin. Evaluation of the expression of DLC-1 might be a good prognostic marker for patients with HCC.

## Background

Liver cancer is one of the most common malignancies in China and the 5-year survival rate of it is very low because of invasion and metastasis in the early stage [1, 2]. Mechanisms of invasion and metastasis are diverse and not yet clear-cut in most cases. So the identification of indicators or markers that may help us to assess tumor behavior of invasion and metastasis are very important for us [3].

Deleted in liver cancer-1 (DLC-1) was first cloned by using subtractive hybridization method in human hepatocellular carcinomas and later it was identified as a breast cancer metastasis suppressor in microarray comparisons between breast cancer cell lines [4, 5]. Then, deletion or promoter methylation of DLC-1 has been described in multiple cancers. DLC-1 is a RhoGAP (GTPase-activating protein) that inhibits/inactivates Rho-dependent signal transduction [4, 6]. While Rho is key factors in cell proliferation, polarity, cytoskeletal remodeling and migration, the aberrant function of their regulators may lead to cell transformation [6, 7]. Data showed that DLC-1 mRNA expression was lost in 95 % of patient with NSCLC tumors tissues and 58 % of NSCLC cell lines, due at least in part through its function as a RhoGAP and thus negatively regulating the expression of RhoA and related RhoB, RhoC [8]. Nevertheless, a detection of DLC-1 as a therapeutic indicator or prognostic markers in liver cancers have litter been elucidated and the role and mechanism of DLC-1 in liver cancer especially in the invasion and metastasis process remains to be fully eclucidated.

There is considerable and growing evidence for the importance of the ROCK gene in actomyosin contractility, focal adhesion assembly, cytokinesis and cell proliferation [9, 10]. ROCK has also been implicated in colorectal [11], breast [12], gastric [13], and hepatocellular cancer metastatic growth [14]. One identified mechanism for ROCK activation in cancer involves the loss of function of the DLC-1, which encodes a GTPase activating protein (RhoGAP) for the RhoA and RhoC small GTPases [15].

Moesin, a member of the ERM (moesin, radixin, ezrin) family of proteins has been reported to be overexpressed in many kinds of cancers [16, 17]. Leroi et al. [18] sowed that moesin not ezrin and radixin was up-regulated in glioblastoma multiforme in comparison to non-malignant brain tissue samples. In addition, He [19] et al. reported that the extracellular small GTPase RhoA/ROCK-2 cascade mediated the increased moesin expression and phosphorylation, however, the relationship between DLC-1 and moesin in liver cancer has not been evaluated before.

In the current study, the relationship between the expression of DLC1 and various clinicopathologic parameters and immunohistochemical markers, such as RhoA, ROCK2 and Moesin were analyzed. Furthermore, the prognostic significance of DLC1 in human HCC was also explored.

## Methods

### Patient specimens

This study was approved by the Ethics Committee of the First Affiliated Hospital of Zhengzhou University (Zhengzhou, Henan, China). Written consents were obtained from all those patients included in this study. A total of 80 formalin-fixed paraffin-embedded liver cancer tissues and the corresponding normal tissues were collected from the archive of the First Affiliated Hospital of Zhengzhou University from 2009 to 2013. None of these patients received radiotherapy or chemotherapy prior to surgery. Briefly, the 80 patients consisted of 19 females and 61 males with ages ranging between 36 and 78 years old (mean age: 65 years old). According to the newer 2010 TNM classification of malignant tumours, liver cancers were staged into 54 I-II and 26 III stages. The histological grade of tumor differentiation was assigned into 61 I--II stage and 29 III-IV stage. Cancer size was calculated by measuring the largest dimension of the cancer specimen. 34 cases localized liver cancers without dissemination in liver and portal vein invasion were viewed as low invasion groups while other 46 cases with dissemination in liver and portal vein invasion were viewed as high.

### Immunohistochemistry

The immunohistochemistry protocols were described previously [20]. Primary antibodies against DLC-1, Rho A, ROCK2 and Moesin were used to detect the corresponding proteins. Briefly, slides were incubated with 1.5 % H_2_O_2_ at room temperature for 20 min to eliminate the endogenous peroxidase activity. Then the slides were incubated with a solution comprising 0.2 % triton-X 100 and 5 % goat serum at room temperature for 2 h to quench immunoglobulin’s non-specific binding. After that, the primary antibodies were added to the slides individually and incubated overnight at 4 °C. Next day, secondary antibody was applied to the slides and incubated at room temperature for 2 h. At last, the immune complexs were visualized by incubating the sections with DAB. The slides were counterstained with hematoxylin and mounted. Negative controls were identically treated with the primary antibodies by PBS.

### Immunohistochemistry evaluation

Immunostained sections were evaluated independently by two pathologists. Immunostaining was evaluated semi-quantitatively by estimating immunoreactivity extent and intensity. Staining extent was rated according to the percentage of positive cells. Samples with no stained tumor cells were rated as 0, those with <10 % of stained tumor cells were rated as 1, those 11–50 % of stained tumor cells were rated as 2, those with 50–75 % of stained tumor cells were rated as 3 and those with >75 % of stained tumor cells were rated as 4. Staining intensity was graded as follows: negative (0), weak (1), moderate (2) and strong (3). The overall scores were the product of staining intensity multiplied the staining extent. An overall staining score of 0–3 and >3 were regarded as negative (−) and positive (+) protein expression, respectively.

### Statistical analyses

The significant difference of DLC-1, Rho A, ROCK2 and moesin gene expression between specimen groups was carried out using the unpaired Student’s *t*-test. The survival rates after tumor removal were calculated by the Kaplan-Meier method, and differences in survival curves were analyzed by the Log-rank tests. Multivariate survival analysis was performed on all the significant characteristics measured by univariate survival analysis through the Cox proportional hazard regression model. Differences were considered statistically significant when the *P*-value was <0.05.

## Results

### The expression of DLC-1, Rho A, ROCK2 and moesin proteins in liver cancer tissues

**Fig. 1:**
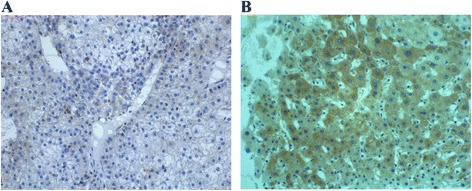
The expression of DLC-1 in HCC tumor and adjacent tissues. **a** Cytoplasm staining of DLC-1 in HCC tumor tissues (×400). **b** Cytoplasm staining of DLC-1 in adjacent normal tissues (×400)

**Fig. 2:**
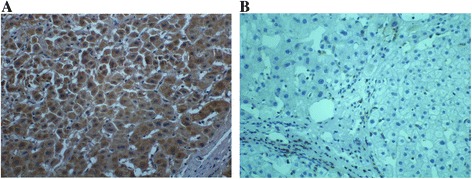
The expression of RhoA in HCC tumor and adjacent tissues. **a** Cytoplasm staining of RhoA in HCC tumor tissues (×400). **b** Cytoplasm staining of RhoA in adjacent normal tissues (×400)

**Fig. 3:**
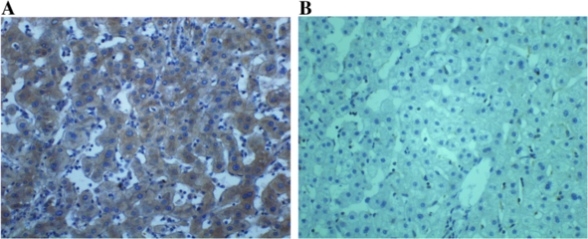
The expression of ROCK2 in HCC tumor and adjacent tissues. **a** ytoplasm staining of ROCK2 in HCC tumor tissues (×400). **b** Cytoplasm staining of ROCK2 in adjacent normal tissues (×400)

**Fig. 4:**
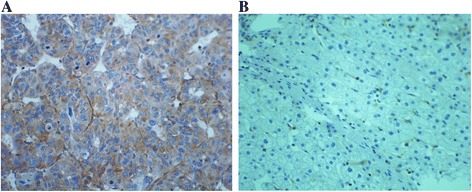
The expression of moesin in HCC tumor and adjacent tissues. **a** Cytoplasm staining of moesin in HCC tumor tissues (×400). **b** Cytoplasm staining of moesin in adjacent normal tissues (×400)

The cellular locations of DLC-1, Rho A, ROCK2 and moesin proteins were all mainly found in the cytoplasm. As shown in Figs. 1a, b; 2a, b; 3a, b and 4a, b, positive staining rates of DLC-1, Rho A, ROCK2 and moesin in liver cancer tissue samples were 38.75, 78.75, 75.00 and 86.25 % respectively, which was significantly different from those in normal mucosa (83.75, 43.75, 40.00 and 66.25 %) (*P* < 0.001, Table 1).

**Table 1:** The expression of DLC-1, RhoA, ROCK2 and moesin in HCC tumor and adjacent tissues

		HCC tissues (*n* = 80)	Adjacent liver tissues (*n* = 80)	*p*
DLC-1	High	31	67	<0.001
	Low	49	13	
RhoA	High	63	35	<0.001
	Low	17	45	
ROCK2	High	60	32	<0.001
	Low	20	48	
moesin	High	69	53	<0.001
	Low	11	27	

### Clinicopathological significance of the expression of DLC-1, Rho A, ROCK2 and moesin proteins in liver cancer tissues

Clinicopathological significance of the expression of DLC-1, Rho A, ROCK2 and moesin proteins in liver cancer tissues are shown in Table 2. We found a negative correlation between DLC-1 expression and Tumor differentiation (*p* < 0.001) and TNM stage (*p* < 0.001).In addition, the expression of DLC-1 in low invasion groups was higher than those in high invasion groups (*p* < 0.001).

**Table 2:** The relationship between DLC-1, RhoA, ROCK2, moesin and clinicopathological variables in HCC tissues

Clinicopatholog-ical variables	DLC1 expression			RhoA expression			Rock2 expression			moesin expression		
	H	L	p	H	L	p	H	L	p	H	L	p
Gender												
Male	23	38	0.731	48	13	0.98	46	15	0.879	55	8	0.29
Female	8	11		15	4		14	5		15	4	
Age (years)												
< 45	9	14	0.958	17	6	0.502	17	6	0.887	19	4	0.548
≥ 45	22	35		46	11		43	14		50	7	
Tumor number												
Singel	20	30	0.767	40	10	0.724	39	11	0.424	43	7	0.933
Multiple	11	19		23	7		21	9		26	4	
Maximal tumor size (cm)												
< 5	19	30	0.995	38	11	0.742	37	13	0.789	43	6	0.623
≥ 5	12	19		25	6		23	7		26	5	
Tumor differentiation												
I-II	12	49	<0.001	36	15	0.018	33	18	<0.001	41	10	0.043
III-IV	19	10		27	2		27	2		28	1	
HBV infection												
No	7	11	0.989	14	4	0.909	13	5	<0.001	15	3	0.683
Yes	24	38		49	13		47	15		54	8	
TNM stage												
I-II	11	43	<0.001	39	15	0.034	37	17	0.002	44	10	0.074
III	20	6		24	2		23	3		25	1	
Portal vein invasion and dissemination												
Yes	8	38	<0.001	39	7	<0.001	40	6	0.004	43	3	0.029
No	23	11		14	20		20	14				

While the expression levels of Rho A, ROCK2 and moesin were positively correlated with Tumor differentiation, and TNM stage. Furthermore, Rho A, ROCK2 and moesin expression levels were significantly higher in HCC cancer patients with high invasion ability.

### Association between DLC-1 and Rho A, ROCK2, moesin expression in HCC cancer tissues

Spearman’s rank correlational analysis revealed a significant negative correlation between the expression levels of DLC-1 and Rho A, ROCK2, moesin (r = −0.963, −0.669, −0.631).

### Survival analysis

**Fig. 5:**
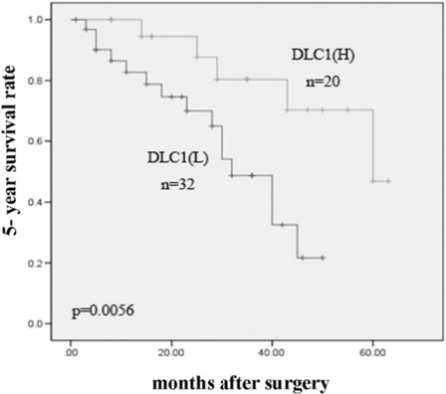
Kaplan-Meier analyses of 5-year survival rates in 52 HCC patients in relation to DLC-1 protein overexpression. Patients with HCC with low DLC-1 expression had lower 5-year *p* < 0.001) survival rates than those with high DLC-1 expression as determined using the Kaplan-Meier method.(H: high DLC-1 expression, L: low DLC-1 expression)

HCC patients with low DLC1 expression had lower disease-free and 5-year survival rates than those with high DLC1 expression as determined using the Kaplan-Meier method (Fig. 5). Univariate Cox regression analysis also identified that clinical variables including gender, tumor number, maximal tumor size, HBV infection, tumor differentiation, TNM stage and portal vein invasion and dissemination were all positively correlated with overall survival. Furthermore, to evaluate the potential of DLC1 expression as an independent prognostic marker for overall survival of HCC, multivariate Cox regression analyses were performed. While the others failed to demonstrate independence, DLC1 expression (HR: 2.234, 95 % CI: 0.937–4.148, *p* < 0.001), tumor differentiation (HR: 3.769, 95 % CI: 1.925–6.989, P = 0.003), and portal vein invasion and dissemination (HR: 1.341,95 % CI: 0.843–2.631, *p* < 0.001) may play a role in predicting the overall survival in HCC (*p* < 0.05).

## Discussion

HCC is one of the most common malignancies worldwide. Despite advances in the detection and treatment of HCC, the mortality rate remains high because of early occurrence of invasion and metastasis [21]. Hence to improve early detection of invasion and metastasis and their molecular characterization in HCC, to develop interventions that are targeted at blocking or reversing the invasion and metastasis process are very important for us.

DLC-1 has been known as tumor suppressor gene in many kinds of cancers including liver, lung, breast, brain, prostate and colon cancers [22, 23]. And re-expression of DLC-1 in cancer cells regulates the structure of actin cytoskeleton and focal adhesions and significantly inhibits cell growth, colony formation and invasion capacity, supporting its role as a tumor suppressor [24]. Thus, DLC-1 has the potential to be a key therapeutic indicator or markers for cancer gene therapy. In the present study, DLC-1 was found to be lower expressed in HCC tissues. DLC-1 positive expression was observed in 31 out of 80 HCC patients. And the lower expression of DLC-1 was correlated negatively with tumor differentiation and TNM stage. More importantly, the lower expression of DLC-1 found to be negatively linked with aggressive clinical behavior. HCC tissues with dissemination in liver and portal vein invasion showed lower DLC-1 expression than those in localized tissues without dissemination in liver and portal vein invasion.

Regarding DLC1 expression, recent reports have shown a growing interest in its prognostic impact in terms of survival. In this study, the prognosis of HCC patients with a low expression of DLC1 was poor, and Cox regression analysis indicated that low expression level of DLC1 was a significant prognostic factor for a poor survival rate of HCC patients. These findings raised the possibility that lower expression of DLC1 could be a factor of worst disease-specific survival, independently of any other variables.

DLC-1 was also named ARHGAP7 because it contains a RhoGTPase activating protein (RhoGAP) domain and these domains can convert the active GTP-bound Rho proteins to the inactive GDP-bound state and negatively regulate Rho GTPases [25]. RhoA is one of the best-known members of RhoGAP. ROCKs were originally isolated as downstream targets of Rho A [26]. RhoA binding with the C-terminal domain of ROCK, forming the Rho A/ROCK pathway, and controls a wide variety of cellular processes dependent on the re-arrangement of the actin cytoskeleton and changes of cell contractility. Yet, ROCK does not directly act on cytoskeletal molecules and ERM (radixin/ezrin/moesin) proteins are emerged as the candidates that likely linking the activation of ROCK and the cytoskeleton reorganization [27, 28]. Moesin is one of the most important and it has been reported that Rho A/ROCK is a typical upstream pathway for the phosphorylation of moesin [29, 30]. In the present study, Rho A and ROCK2 expression were found to negatively correlated with DLC-1 expression. Rho A and ROCK2 expression were identified in 63 and 60 of the HCC cases. Expression of Rho A and ROCK2 were all correlated positively tumor differentiation and TNM stage dissemination in liver and portal vein invasion. Furthermore, positive expression of Rho A and ROCK2 were also linked with dissemination in liver and portal vein invasion of HCC tissues. These data indicated that DLC-1, Rho A and ROCK2 perhaps together played an important role in the invasion and metastasis of HCC, and that DLC-1 perhaps played as a tumor suppressor by inhibiting the activation of Rho A/ROCK2 pathway . Furthermore, we found that the expression of phosphorylation of moesin was negatively correlated with the expression of DLC-1. Positive expression of phosphorylation of moesin was all correlated positively with tumor differentiation, TNM stage and dissemination in liver and portal vein invasion. So DLC-1 perhaps exerted its effects in invasion in HCC by regulating the expression of moesin through the RhoA/ROCK pathway.

Regarding DLC1 expression, recent reports have shown a growing interest in its prognostic impact in terms of survival. In this study, the prognosis of HCC patients with a low expression of DLC1 was poor, and Cox regression analysis indicated that low expression level of DLC1 was a significant prognostic factor for a poor survival rate of HCC patients. These findings raised the possibility that lower expression of DLC1 could be a factor of worst disease-specific survival, independently of any other variables.

## Conclusion

DLC-1 appeared to also play as a tumor suppressor gene in HCC. And DLC-1 might work its way by negatively regulating the expression of RhoA/ROCK2/ moesin. DLC-1 itself could be a direct indicator or marker for the invasion and prognosis of HCC.
